# Comparison of Tocilizumab vs Baricitinib in Clinical Outcomes Among Hospitalized Patients With COVID-19: Experience From a Public Hospital System in New York City

**DOI:** 10.1093/ofid/ofad426

**Published:** 2023-08-09

**Authors:** Subin Sunny, Ami Tran, Jennifer Lee, Marie Abdallah, Nimra Chaudhry, John Quale

**Affiliations:** Division of Infectious Diseases, Department of Medicine, NYC Health + Hospitals/Kings County, Brooklyn, New York, USA; Division of Infectious Diseases, Department of Medicine, NYC Health + Hospitals/Kings County, Brooklyn, New York, USA; Division of Infectious Diseases, Department of Medicine, NYC Health + Hospitals/Kings County, Brooklyn, New York, USA; Division of Infectious Diseases, Department of Medicine, NYC Health + Hospitals/Kings County, Brooklyn, New York, USA; Department of Pharmacy, NYC Health + Hospitals/Queens, Queens, New York, USA; Division of Infectious Diseases, Department of Medicine, NYC Health + Hospitals/Kings County, Brooklyn, New York, USA

**Keywords:** COVID-19, baricitinib, tocilizumab

## Abstract

**Background:**

Tocilizumab and baricitinib are immunomodulators that have been repurposed for the treatment of coronavirus disease 2019 (COVID-19). Whether one medication should be preferred over the other has not been established.

**Methods:**

This multicenter retrospective cohort study comprised hospitalized patients with COVID-19 who received either tocilizumab or baricitinib. The primary outcome was improvement in respiratory status (at least 1-point reduction on the respiratory ordinal scale) at day 7 and up to day 28. Secondary outcomes included mortality, disposition, deep vein thrombosis, pulmonary embolism, or positive blood culture. Outcomes were stratified by baseline respiratory status and variant-predominating periods. Results were reported for the overall and propensity-matched cohorts.

**Results:**

A total of 921 patients received tocilizumab and 638 received baricitinib. The propensity-matched cohort included 597 patients in each group. At day 7 in the overall and propensity-matched cohorts, significantly more patients had improvement in respiratory status in the baricitinib group. These improvements were seen in patients requiring supplemental oxygen and noninvasive ventilation/high-flow oxygen but not in patients requiring mechanical ventilation. Favorable outcomes with baricitinib were observed during the Alpha and Omicron periods. By day 28, there were no differences in the changes of respiratory status for the treatment groups in either cohort. Also, no differences were seen in mortality, disposition, development of deep vein thrombosis/pulmonary embolism, or bloodstream infections.

**Conclusions:**

Baricitinib treatment was associated with more favorable respiratory improvement at day 7 when compared with tocilizumab, but no differences were observed up to day 28.

## Teaser

In this multicenter retrospective cohort study, we observed that among patients hospitalized with COVID-19, those treated with baricitinib had more favorable respiratory improvement by day 7 when compared with tocilizumab, but no differences were observed up to day 28.

Coronavirus disease 2019 (COVID-19), caused by the novel severe acute respiratory syndrome coronavirus 2 (SARS-CoV-2), was first identified in December 2019 in a cluster of patients with pneumonia in Wuhan, China, and has since rapidly spread, resulting in a global pandemic [1]. Patients with COVID-19 can experience a range of clinical manifestations, from asymptomatic to critical illness. The early clinical course of the disease is thought to be driven by viral replication, while the later course may be driven by a dysregulated immune or inflammatory response. Remdesivir has been approved by the Food and Drug Administration for treatment of COVID-19 and has been shown to be beneficial during the early course of the disease, which is driven by viral replication, while corticosteroids have been the mainstay of treatment for the later course of the disease due to its anti-inflammatory effects. Other immunomodulators have been studied for the treatment of COVID-19: tocilizumab and baricitinib are two such immunomodulators that have shown clinical benefit in the treatment of COVID-19 [2].

Tocilizumab is a recombinant humanized anti–interleukin 6 receptor monoclonal antibody that was approved by the Food and Drug Administration for use in patients with rheumatologic disorders and cytokine release syndrome induced by chimeric antigen receptor T-cell therapy [2]. Two large randomized controlled trials (REMAP-CAP, RECOVERY) showed a survival benefit with tocilizumab, mainly in patients who exhibited rapid respiratory decompensation associated with a systemic inflammatory response [3, 4]. In contrast, in the REMDACTA trial—conducted in patients with COVID-19 who required noninvasive ventilation (NIV) or high-flow oxygen support—use of tocilizumab did not show a reduction in 28-day mortality [5].

Baricitinib is an oral Janus kinase (JAK) inhibitor, selective for JAK1 and JAK2, which can modulate downstream inflammatory responses [2]. The RECOVERY and COV-BARRIER trials showed a survival benefit in patients with COVID-19 treated with baricitinib plus dexamethasone, which was most pronounced among patients receiving high-flow nasal canula or NIV [6, 7]. Additionally, data from the ACTT-2 and ACTT-4 trials supported the potential clinical benefit of baricitinib [8, 9].

In addition to dexamethasone, tocilizumab or baricitinib is recommended in the National Institutes of Health’s COVID-19 treatment guidelines for patients with rapidly increasing oxygen needs and systemic inflammation and for patients requiring high-flow nasal canula, NIV, mechanical ventilation, or extracorporeal membrane oxygenation; however, neither option is preferred over the other [2]. Limited data exist to compare outcomes between the anti-inflammatory treatment options. The objective of our study was to compare outcomes in patients hospitalized with COVID-19 treated with tocilizumab or baricitinib.

## METHODS

This multicenter retrospective cohort study consisted of patients admitted to the 11 tertiary care academic hospitals composing the New York City Health and Hospitals System. These hospitals serve patients primarily of low socioeconomic status in the boroughs of Bronx, Brooklyn, Manhattan, and Queens. Patients were included if they were admitted and received tocilizumab or baricitinib for treatment of COVID-19 between December 2020 and March 2022. Patients were excluded if they were administered tocilizumab and baricitinib, if either medication was used for an indication other than COVID-19, or if they received subcutaneous tocilizumab.

Data were collected via retrospective chart review. Baseline characteristics were collected as follows: age, date of admission, date of discharge, gender, race/ethnicity, COVID-19 vaccination status, comorbidities (diabetes, hypertension, lung disease, chronic kidney disease), weight, body mass index, level of service at initiation of treatment, serum creatinine (SCr) and C-reactive protein at start of treatment, date of first dose of tocilizumab or baricitinib, duration of treatment (baricitinib), number of doses (tocilizumab) and if patients received remdesivir (during the same hospitalization), corticosteroids (within 7 days of first dose), or vasopressors (at time of treatment initiation). Respiratory status, based on the National Institute of Allergy and Infectious Diseases Ordinal Scale (scores, OS1–OS8; Table 1), was collected at days 0, 7, 14, and 28, with day 0 being the first day of treatment with tocilizumab or baricitinib. Data were also collected regarding the presence of new deep vein thrombosis (DVT), pulmonary embolism (PE), or positive blood culture (all within 28 days of first dose), as well as final disposition of the patient (home, transferred to another health care facility, or death).

**Table 1.** Baseline Demographics of Patients Who Received Tocilizumab and Baricitinib^a^

	Overall			Matched		
	Tocilizumab (n = 921)	Baricitinib (n = 638)	*P* Value	Tocilizumab (n = 597)	Baricitinib (n = 597)	*P* Value
Age, y	61.7 ± 16.6	63.1 ± 16.1	.101	62.2 ± 16.4	62.8 ± 16.1	.48
Male	530 (58)	318 (50)	.003	316 (53)	310 (52)	.728
Race/ethnicity						
Hispanic	302 (33)	291 (45)	<.001	215 (36)	266 (45)	.003
Black/African American	284 (31)	180 (28)	.265	165 (28)	175 (29)	.521
White	126 (14)	71 (11)	.136	85 (14)	65 (11)	.081
Asian	121 (13)	46 (7)	<.001	64 (11)	46 (8)	.072
Other	88 (10)	50 (8)	.24	68 (11)	45 (8)	.023
Comorbidities						
Diabetes mellitus	359 (39)	265 (42)	.311	235 (39)	243 (41)	.637
Hypertension	519 (56)	391 (61)	.052	345 (58)	361 (60)	.346
Lung disease	137 (15)	146 (23)	<.001	106 (18)	125 (21)	.164
Chronic kidney disease	90 (10)	31 (5)	<.001	30 (5)	30 (5)	>.99
Vaccination status						
Unvaccinated	659 (72)	513 (80)	<.001	464 (78)	485 (81)	.132
Level of service						
Medical/surgical	580 (63)	406 (64)	.79	383 (64)	377 (63)	.718
ICU	341 (37)	232 (36)	.79	214 (36)	220 (37)	.718
Steroids	917 (99.5)	637 (99.8)	.341	596 (99.8)	597 (100)	.317
RDV	826 (90)	618 (97)	<.001	582 (97)	577 (97)	.391
Vasopressors	66 (7)	51 (8)	.542	45 (8)	45 (8)	>.99
Weight, kg	86.8 ± 26.2	87.7 ± 27.4	.533	87.6 ± 27.4	87.5 ± 26.7	.97
Body mass index, kg/m^2^	31.1 ± 8.3	31.9 ± 9.2	.097	31.6 ± 8.9	31.6 ± 8.8	>.99
Serum creatinine, mg/dL	1.3 ± 1.4	0.99 ± 0.58	<.001	1.0 ± 0.7	1.0 ± 0.6	.4
Period						
Wild type	324 (35)	290 (45)	<.001	217 (36)	292 (49)	<.001
Alpha variant	221 (24)	48 (8)	<.001	119 (20)	44 (7)	<.001
Delta variant	253 (27)	170 (27)	.719	188 (31)	169 (28)	.23
Omicron variant	123 (13)	130 (20)	<.001	73 (12)	92 (155)	.111
Baseline respiratory status^b^						
OS3	1 (0.1)	0	.405	0	0	…
OS4	39 (4)	6 (1)	<.001	12 (2)	6 (1)	.154
OS5	281 (31)	174 (27)	.167	170 (28)	172 (29)	.898
OS6	501 (54)	361 (57)	.393	347 (58)	334 (56)	.447
OS7	99 (11)	97 (15)	.009	68 (11)	85 (14)	.141

Abbreviations: ECMO, extracorporeal membrane oxygenation; ICU, intensive care unit; NIV, noninvasive ventilation; RDV, remdesivir.

a Data are presented as No. (%) or mean ± SD.

b Respiratory status based on the National Institute of Allergy and Infectious Diseases Ordinal Scale: OS1, not hospitalized, no limitation on activities; OS2, not hospitalized, limitations on activities and/or requiring home oxygen; OS3, hospitalized, no supplemental oxygen, no longer requires ongoing medical care; OS4, hospitalized, no supplemental oxygen, requiring ongoing medical care; OS5, hospitalized, requiring supplemental oxygen; OS6, hospitalized, receiving NIV or high-flow oxygen devices; OS7, hospitalized, receiving invasive mechanical ventilation or ECMO; OS8, death.

### Outcomes

The primary outcome was improvement in respiratory status at day 7, defined by at least a 1-point reduction on the respiratory scale, and the last known respiratory status up to days 14 and 28. Secondary outcomes included (1) improvement in respiratory status with at least a 2-point reduction on the scale at day 7 and up to day 28, (2) worsening in respiratory status by 1 or 2 points at day 7 and up to day 28, (3) in-hospital mortality, (4) disposition at discharge, and (5) development of DVT/PE or positive blood culture within 28 days of first dose. Outcomes were also analyzed according to baseline respiratory status. In addition, outcomes were evaluated by the periods during which there was a predominant SARS-CoV-2 strain circulating in New York City: wild type (March 2020–March 2021), Alpha (April 2021–June 2021), Delta (July 2021–December 2021), and Omicron (January 2022–March 2022). Results were reported for the overall cohort as well as the propensity-matched cohorts.

### Statistical Analysis

Differences in baseline demographics and outcomes were compared between the tocilizumab and baricitinib groups with the chi-square test for categorical variables and the *t* test for continuous variables. Propensity score matching was done through SPSS Version 28.0.1.1 (15) (IBM) to match patients in either group with a caliper of 0.2 times the standard deviation of the propensity score logit. The following covariates were used to calculate propensity scores: age, SARS-CoV-2 variant period, gender, race, body mass index, vaccination status, comorbidities, level of service, and SCr, as well as receipt of steroids, remdesivir, and vasopressors.

This study was approved by the Institutional Review Board at SUNY–Downstate Medical Center and the System to Track and Approve Research at NYC Health and Hospitals.

## RESULTS

A total of 1596 patients were reviewed for inclusion. Thirty-seven patients were excluded: 22 received tocilizumab and baricitinib, and 15 received either medication for another indication. Baseline demographics are summarized in Table 1. The overall cohort included 921 patients in the tocilizumab group and 638 in the baricitinib group. There were significantly more males and more Asian patients in the tocilizumab group, while the baricitinib group had more Hispanic patients. More patients in the baricitinib group had a history of lung disease, while more in the tocilizumab group had chronic kidney disease. Most patients in both groups were unvaccinated, with a higher percentage in the baricitinib group. Among patients who received vaccinations prior to infection with COVID-19, receipt of mRNA vaccines was the most common. In the tocilizumab group, 23% of patients received at least 1 dose of an mRNA vaccine, as compared with 16% in the baricitinib group; 3% in each group received at least 1 dose of the Johnson & Johnson vaccine. Patients in the baricitinib group were more likely to receive remdesivir. The tocilizumab group had a higher baseline SCr and C-reactive protein at day 0. In terms of respiratory status, more patients in the tocilizumab group had a baseline status of OS4, while more in the baricitinib group had a baseline status of OS7.

The propensity-matched cohort was more balanced, although some differences did still persist (Table 1). For example, among the 597 patients in each group, there were more Hispanic patients in the baricitinib group. For each cohort, the median time from positive SARS-CoV-2 test to start of either treatment was 2 days. For the overall and propensity-matched groups, the mean duration of baricitinib treatment was 7 days. Similarly, 97% of patients in each group received 1 dose of tocilizumab.

Outcome results are reported in Table 2. We observed similar outcomes in the overall and propensity-matched cohorts. The median length of stay was the same (13 days) for the groups in the overall cohort and the propensity-matched cohort. No significant differences were seen when comparing changes in respiratory status up to day 14 and day 28 between the groups in either cohort. At day 7, more patients had improvement in respiratory status (1 and 2 points) in the baricitinib group, which was seen in the overall and matched cohorts. More patients had worsening by 1 point on the respiratory scale in the tocilizumab group in both cohorts. No differences were seen in disposition (including mortality), development of DVT/PE, or bloodstream infections.

**Table 2.** Outcomes of Patients Treated With Tocilizumab and Baricitinib in the Overall and Propensity-Matched Cohorts

	Overall, No. (%)			Matched, No. (%)		
Outcome	Tocilizumab (n = 921)	Baricitinib (n = 638)	*P* Value	Tocilizumab (n = 597)	Baricitinib (n = 597)	*P* Value
Day 28 (final)^a^						
Point improvement						
≥1	551 (60)	391 (61)	.562	346 (58)	365 (61)	.263
≥2	520 (56)	376 (59)	.331	325 (54)	351 (59)	.129
Point worsening						
≥1	324 (35)	220 (34)	.777	219 (37)	209 (25)	.546
≥2	227 (25)	141 (22)	.244	149 (25)	134 (22)	.307
Day 7^a^						
Point improvement						
≥1	347 (38)	287 (45)	.004	220 (37)	265 (44)	.008
≥2	214 (23)	183 (29)	.015	135 (23)	169 (28)	.024
Point worsening						
≥1	248 (27)	127 (20)	.001	155 (26)	117 (20)	.009
≥2	72 (8)	34 (5)	.055	45 (8)	31 (5)	.097
At discharge						
Disposition						
Home	512 (56)	347 (54)	.639	325 (54)	325 (54)	>.99
Dead	317 (34)	211 (33)	.581	216 (36)	199 (33)	.302
Transferred	92 (10)	80 (13)	.114	56 (9)	73 (12)	.113
Days 1–28						
DVT/PE	70 (8)	42 (7)	.444	49 (8)	40 (7)	.321
Positive blood culture	79 (9)	65 (10)	.28	53 (9)	62 (10)	.377

Abbreviations: DVT, deep vein thrombosis; PE, pulmonary embolism.

a Respiratory status at day 7/28 based on the National Institute of Allergy and Infectious Diseases Ordinal Scale.

Outcomes were evaluated by baseline respiratory score as well (Tables 3–5). For patients with a starting status of OS5, more patients in the baricitinib group had improvement in respiratory status by 1 and 2 points by day 28, which was statistically significant in the matched cohort and trended toward significance in the overall cohort. This difference favoring baricitinib was seen for respiratory improvement at day 7 in the overall cohort and for 1-point improvement in the matched cohort. Patients in the baricitinib group had a significantly lower mortality in the matched population, with a trend toward significance in the overall population. For patients with a baseline of OS6, significant differences in respiratory improvement and worsening at day 7 favoring the baricitinib group were observed in the overall cohort but not in the matched cohort. Minimal differences were seen in patients who were on mechanical ventilation at the start of treatment, except for a significantly greater number of patients in the tocilizumab group who died by day 28.

**Table 3.** Outcomes of the Treatment Groups With a Baseline Respiratory Score of OS5 (Requiring Supplemental Oxygen)

	Overall, No. (%)			Matched, No. (%)		
Respiratory Status^a^	Tocilizumab (n = 281)	Baricitinib (n = 174)	*P* Value	Tocilizumab (n = 158)	Baricitinib (n = 158)	*P* Value
Day 28 (final)						
Point improvement						
≥1	227 (81)	152 (87)	.068	123 (78)	139 (88)	.017
≥2	225 (80)	151 (87)	.067	122 (77)	138 (87)	.018
Point worsening						
≥1	47 (17)	21 (12)	.176	31 (20)	19 (12)	.064
≥2	45 (16)	19 (11)	.129	29 (18)	17 (11)	.056
Day 7						
Point improvement						
≥1	136 (48)	105 (60)	.013	77 (49)	95 (60)	.042
≥2	110 (39)	88 (51)	.017	66 (42)	80 (51)	.114
Point worsening						
≥1	70 (25)	30 (17)	.055	41 (26)	27 (17)	.055
≥2	16 (6)	14 (8)	.326	10 (6)	14 (9)	.396
Dead	45 (16)	17 (10)	.059	29 (18)	15 (9)	.023

a Respiratory status based on the National Institute of Allergy and Infectious Diseases Ordinal Scale.

**Table 4.** Outcomes of the Treatment Groups With a Baseline Respiratory Score of OS6 (Requiring Noninvasive Ventilation/High-Flow Oxygen)

	Overall, No. (%)			Matched, No. (%)		
Respiratory Status^a^	Tocilizumab (n = 501)	Baricitinib (n = 361)	*P* Value	Tocilizumab (n = 330)	Baricitinib (n = 330)	*P* Value
Day 28 (final)						
Point improvement						
≥1	261 (52)	200 (55)	.337	184 (56)	177 (54)	.584
≥2	236 (47)	186 (52)	.201	166 (50)	164 (50)	.876
Point worsening						
≥1	220 (44)	150 (42)	.49	132 (40)	143 (43)	.385
≥2	177 (35)	122 (34)	.641	107 (32)	115 (35)	.51
Day 7						
Point improvement						
≥1	174 (35)	158 (44)	.007	122 (37)	139 (42)	.176
≥2	75 (15)	74 (20)	.034	53 (16)	65 (20)	.223
Point worsening						
≥1	146 (29)	83 (23)	.044	77 (23)	77 (23)	>.99
≥2	51 (10)	20 (6)	.015	30 (9)	18 (5)	.072
Dead	209 (42)	141 (39)	.433	125 (38)	133 (40)	.523

a Respiratory status based on the National Institute of Allergy and Infectious Diseases Ordinal Scale.

**Table 5.** Outcomes of the Treatment Groups With a Baseline Respiratory Score of OS7 (Requiring Mechanical Ventilation)

	Overall, No. (%)			Matched, No. (%)		
Respiratory Status^a^	Tocilizumab (n = 99)	Baricitinib (n = 97)	*P* Value	Tocilizumab (n = 67)	Baricitinib (n = 67)	*P* Value
Day 28 (final)						
Point improvement						
≥1	31 (31)	33 (34)	.686	22 (33)	21 (31)	.853
≥2	28 (28)	33 (34)	.386	19 (28)	21 (31)	.706
Point worsening						
≥1	52 (53)	33 (34)	.009	36 (54)	35 (52)	.863
≥2	…	…	…	…	…	…
Day 7						
Point improvement						
≥1	22 (22)	19 (20)	.65	14 (21)	12 (18)	.662
≥2	14 (14)	16 (16)	.647	9 (13)	9 (13)	>.99
Point worsening						
≥1	16 (16)	14 (14)	.737	11 (16)	10 (15)	.812
≥2	…	…	…	…	…	…
Dead	59 (60)	53 (55)	.483	41 (61)	38 (57)	.598

a Respiratory status based on the National Institute of Allergy and Infectious Diseases Ordinal Scale.

Outcomes were evaluated by the period during which the patients received treatment reflecting the predominant variant present in New York City (Tables 6–9). No outcome differences were seen when the wild type virus and Delta variant were predominant. During the period in which the Alpha variant was predominant, patients tended to improve more in the baricitinib group by day 7, and a difference in mortality was seen in the overall cohort (tocilizumab 26% vs baricitinib 13%, *P* = .04). There were significant differences seen for respiratory improvement and worsening in the overall cohort by days 7 and 28 during the Omicron period, favoring the baricitinib group. A lower mortality rate in the baricitinib group was also observed in the overall cohort (tocilizumab 49% vs baricitinib 39%, *P* = .001). In the matched cohort, significant outcome differences were seen at day 7 but not the final respiratory status.

**Table 6.** Respiratory Outcomes at Day 7 of the Treatment Groups During the Wild Type and Alpha Periods

	Wild Type, No. (%)			Alpha, No. (%)		
Respiratory Status^a^	Tocilizumab	Baricitinib	*P* Value	Tocilizumab	Baricitinib	*P* Value
Overall cohort	324	290		221	48	
Point improvement						
≥1	125 (39)	116 (40)	.719	81 (37)	29 (61)	.002
≥2	78 (24)	84 (29)	.17	56 (25)	16 (33)	.257
Point worsening						
≥1	80 (25)	72 (25)	.969	71 (32)	3 (6)	<.001
≥2	26 (8)	17 (6)	.295	15 (7)	1 (2)	.212
Matched cohort						
Point improvement	227	227		41	41	
≥1	87 (38)	91 (40)	.701	16 (39)	25 (61)	.047
≥2	57 (25)	67 (30)	.292	10 (24)	14 (34)	.332
Point worsening						
≥1	61 (27)	57 (25)	.669	10 (24)	2 (5)	.012
≥2	21 (9)	15 (7)	.297	3 (7)	1 (2)	.305

a Respiratory status based on the National Institute of Allergy and Infectious Diseases Ordinal Scale.

**Table 7.** Respiratory Outcomes at Day 7 of the Treatment Groups During the Delta and Omicron Periods

	Delta, No. (%)			Omicron, No. (%)		
Respiratory Status^a^	Tocilizumab	Baricitinib	*P* Value	Tocilizumab	Baricitinib	*P* Value
Overall cohort	253	170		123	130	
Point improvement						
≥1	103 (41)	76 (45)	.717	38 (31)	66 (51)	.001
≥2	65 (26)	42 (25)	.974	15 (12)	41 (32)	<.001
Point worsening						
≥1	56 (22)	31 (18)	.623	41 (33)	21 (16)	.001
≥2	13 (5)	12 (7)	.714	18 (15)	4 (3)	.001
Matched cohort	161	161		83	83	
Point improvement						
≥1	69 (43)	70 (43)	.910	23 (28)	36 (43)	.035
≥2	39 (24)	37 (23)	.793	9 (11)	20 (24)	.025
Point worsening						
≥1	31 (19)	30 (19)	.887	25 (30)	15 (18)	.07
≥2	8 (5)	12 (7)	.356	10 (12)	2 (2)	.016

a Respiratory status based on the National Institute of Allergy and Infectious Diseases Ordinal Scale.

**Table 8.** Respiratory Outcomes at Day 28 of the Treatment Groups During the Wild Type and Alpha Periods

	Wild Type, No. (%)			Alpha, No. (%)		
Respiratory Status^a^	Tocilizumab	Baricitinib	*P* Value	Tocilizumab	Baricitinib	*P* Value
Overall cohort	324	290		221	48	
Point improvement						
≥1	182 (56)	162 (56)	.938	149 (67)	39 (81)	.058
≥2	174 (54)	157 (54)	.914	142 (64)	37 (77)	.088
Point worsening						
≥1	121 (37)	118 (41)	.396	67 (30)	7 (15)	.027
≥2	84 (26)	71 (24)	.681	50 (23)	4 (48)	.025
Matched cohort	227	227		41	41	
Point improvement						
≥1	119 (52)	126 (56)	.51	27 (66)	33 (80)	.135
≥2	113 (50)	123 (54)	.348	25 (61)	31 (76)	.154
Point worsening						
≥1	93 (41)	93 (41)	>.99	14 (34)	6 (15)	.04
≥2	67 (30)	56 (25)	.245	6 (15)	4 (10)	.50

a Respiratory status based on the National Institute of Allergy and Infectious Diseases Ordinal Scale.

**Table 9.** Respiratory Outcomes at Day 28 of the Treatment Groups During the Delta and Omicron Periods

	Delta, No. (%)			Omicron, No. (%)		
Respiratory Status^a^	Tocilizumab	Baricitinib	*P* Value	Tocilizumab	Baricitinib	*P* Value
Overall cohort	253	170		123	130	
Point improvement						
≥1	162 (64)	108 (64)	.916	58 (47)	82 (63)	.011
≥2	151 (60)	101 (59)	.955	53 (43)	81 (62)	.002
Point worsening						
≥1	74 (29)	56 (33)	.42	62 (50)	39 (30)	<.001
≥2	47 (19)	39 (23)	.274	46 (37)	27 (21)	.004
Matched cohort	161	161		83	83	
Point improvement						
≥1	102 (63)	99 (61)	.73	42 (51)	48 (58)	.35
≥2	96 (60)	93 (58)	.734	38 (46)	47 (57)	.162
Point worsening						
≥1	50 (31)	57 (35)	.408	38 (46)	27 (33)	.08
≥2	33 (20)	39 (24)	.422	29 (35)	21 (25)	.176

a Respiratory status based on the National Institute of Allergy and Infectious Diseases Ordinal Scale.

## DISCUSSION

A number of studies have been conducted that evaluate the difference between tocilizumab and baricitinib for treatment of severe COVID-19. An open-label randomized controlled trial compared outcomes between baricitinib and tocilizumab, which showed that baricitinib was noninferior to tocilizumab for the composite outcome of mechanical ventilation or death by day 28 and time to discharge by day 28 [10]. The majority of the additional studies have been smaller retrospective cohort studies, which all showed no difference in the outcomes evaluated between baricitinib and tocilizumab [11–17]. In addition, a prospective real-world study and a systematic review and meta-analysis reported no differences between the treatments [18, 19]. One meta-analysis reported better 28-day mortality data with baricitinib when compared with tocilizumab [20]. Last, one study evaluated the impact of adding baricitinib to standard of care, which included tocilizumab and corticosteroids but found no difference in mortality [21]. Despite no difference, this therapeutic approach may warrant further investigation.

In this propensity-matched retrospective cohort study, we found no differences between tocilizumab and baricitinib treatment among patients with COVID-19 in terms of respiratory improvement or worsening by day 28. This correlates with most of the published data available to date, although we did find a noticeable benefit of baricitinib when evaluating outcomes at day 7. This difference was seen in the overall cohort as well and seemed to be driven by patients who had a baseline respiratory status of OS5 or OS6. We found no differences in disposition at discharge or in development of DVT/PE or positive blood culture within 28 days of starting therapy.

Our results indicate that baricitinib provides greater short-term benefit in patients requiring oxygen therapy but not to the point of requiring mechanical ventilation. The use of remdesivir and corticosteroids in the matched cohort were nearly universal; therefore, these were unlikely to be confounding variables. The reason for a more favorable short-term response for baricitinib may be due to differences in the mechanism of action of the 2 drugs. Tocilizumab specifically inhibits interleukin 6, while baricitinib is thought to inhibit multiple inflammatory pathways. The broader anti-inflammatory effect may be the driving factor for the outcome differences at day 7. It is plausible that differences in the pharmacokinetic profiles of the 2 medications may play a role. Tocilizumab reaches peak levels quickly after intravenous administration and starts to decline slowly for the next few days to weeks, depending on the dose given [22, 23]. Baricitinib is administered orally but continuously over multiple days. The continuous administration of baricitinib may lead to more consistent levels of the drug to maintain its anti-inflammatory effect. In contrast, the declining tocilizumab levels following a single dose may not adequately suppress the inflammatory response, leading to negative outcomes in patients with severe COVID-19. However, this is difficult to interpret as the relationship between tocilizumab levels and response in patients with COVID-19 has not been clearly defined [24]. Given that a majority of patients received 1 dose, it may be plausible that a second dose of tocilizumab after at least 8 hours may improve outcomes [2]. Similarly, the mean duration of treatment with baricitinib was 7 days, which is the time point that significant differences in respiratory improvement and worsening were seen. As baricitinib can be used for up to 14 days, a longer duration of baricitinib treatment may be needed to observe more sustained favorable outcomes at day 28.

When subgroups were evaluated by the predominant variant circulating in the area, significant differences in outcomes were seen only during the periods when the Alpha and Omicron variants were predominant, also favoring baricitinib. However, these differences were less pronounced in the matched cohort as compared with the overall cohort. The virulence of the different variants may have been a factor in the outcomes that we observed. This is difficult to interpret during the Alpha period, as there were vastly more patients in the tocilizumab group and the matched cohort had a much smaller sample size. The differences seen during the Omicron period may indicate that baricitinib is more effective at suppressing the inflammatory response caused by this variant, possibly due to a decreased virulence of this strain; however, given the smaller sample size, it is difficult to make this determination.

Strengths of our study include the large patient population and multicenter nature of the study. Racial and ethnic groups that are typically underrepresented in studies (ie, Hispanic, Black, and Asian patients) were highly represented in our cohort, which strengthens the generalizability of our results. These groups have been more negatively affected by the pandemic. To our knowledge, this is the largest study to evaluate the difference in outcomes between tocilizumab and baricitinib. This is also the first study to distinguish tocilizumab and baricitinib using time points reflecting the predominant circulating virus variants.

The study had a number of limitations. Given that this was a retrospective study, we were limited in our ability to control for confounding variables; however, the results from the propensity-matched groups correlated with those of the overall cohort. Although retrospective studies have their inherent limitations, it is recognized that current guideline recommendations for use of these agents are made with an overall lack of direct comparisons; therefore, retrospective studies are still important to provide data on comparisons between these agents [25]. We also did not evaluate laboratory data to further explore the safety and efficacy of these 2 treatment options. Practice variations may have existed among the different hospitals, leading to discrepancies in criteria for use of baricitinib or tocilizumab, which may have contributed to the heterogeneity of the overall patient population and thus affected outcomes. Dosing of the 2 drugs was not evaluated, and variations in dosing practices may have been a confounding factor.

In summary, short-term respiratory outcomes for patients with COVID-19 treated with baricitinib were more favorable than for those treated with tocilizumab in our propensity-matched cohort, although no differences in outcomes were noted in overall or respiratory outcomes up to 28 days. Ultimately, the clinical significance of these results is uncertain as, to our knowledge, short-term respiratory improvement has not been associated with improvement in short- or long-term clinical outcomes. Further research is needed to elucidate any potential differences between these treatments and whether early respiratory improvement has any effect on long-term clinical outcomes. Finally, the optimal duration of baricitinib and dosing of tocilizumab may be important areas of research that warrant further evaluation.

## Notes

***Patient consent statement.*** The design of the study does not require obtaining patient consent and was approved by the Institutional Review Board at SUNY–Downstate Medical Center and the System to Track and Approve Research at NYC Health and Hospitals.
